# Artery of Percheron Stroke: A Case Report With a Diagnostic Challenge

**DOI:** 10.7759/cureus.21939

**Published:** 2022-02-05

**Authors:** Neha Phate, Twinkle Pawar, Amol Andhale, Rohan Kumar Singh, Dhruv Talwar, Sourya Acharya, Samarth Shukla, Sunil Kumar

**Affiliations:** 1 Department of Medicine, Jawaharlal Nehru Medical College, Datta Meghe Institute of Medical Sciences (Deemed to be University), Wardha, IND; 2 Department of Radiodiagnosis, Jawaharlal Nehru Medical College, Datta Meghe Institute of Medical Sciences (Deemed to be University), Wardha, IND; 3 Department of Pathology, Jawaharlal Nehru Medical College, Datta Meghe Institute of Medical Sciences (Deemed to be University), Wardha, IND

**Keywords:** artery, midbrain, brain, tumour, thalamus

## Abstract

Artery of Percheron (AOP) is a unique anatomical variant of blood supply to the paramedian thalamus and also to the rostral part of the midbrain. It arises from the P1 part of the posterior cerebral artery. Obstruction of this artery accounts for the infarction of the bilateral thalamus with or without the involvement of the midbrain. Symptoms of artery of Percheron infarction may differ with respect to the portion of the brain it supplies and its different anatomical variations. The various symptoms include memory loss, altered consciousness, vertical gaze palsy, and others. Diagnosis is difficult due to a variety of clinical presentations and differential diagnoses like viral infections or tumors. Artery of Percheron infarction rarely occurs, and early diagnosis is a challenge as it is often missed on a conventional CT scan and even on an MRI scan of the brain. Delay in diagnosis and initiation of treatment must be avoided in such cases. We report a case of this 57-year-old male who had vertical gaze palsy and irrelevant talks, which was evaluated further and found to be the artery of Percheron infarct on MRI brain and treated with antiplatelets after which the symptoms of the patient ameliorated, and he was discharged after five days of admission.

## Introduction

The thalamus regulates awareness, sleep, and alertness by acting as a relay spot for sensory and motor mechanisms. With a huge amount of feeding arteries, the thalamus and the midbrain have a complicated blood supply. Various perforating branches arising from the posterior communicating artery and the posterior cerebral artery furnish this supply. Small arteries, coming from the posterior communicating artery with segment P1 and segment P2 of the posterior communicating artery, supply the thalamus. The vascular supply to the thalamus is traditionally divided into four regions: anterior, paramedian, inferolateral, and posterior. There are substantial differences and intersections. Incidence of acute infarct of artery of Percheron (AOP) is about 0.1-2% of total ischemic strokes and 4-35 % of total thalamic strokes [1].

The most prevalent artery of Percheron infarction patterns are the bilateral paramedian thalamic with midbrain infarction (43%), bilateral paramedian thalamic alone without midbrain involvement (38%), and bilateral paramedian thalamic infarction with anterior thalamic and midbrain involvement (38%) [2]. It's a type of posterior flow infarction.

Ischemia in the region of an artery of Percheron generally shows up along with primary symptoms that are observed in subjects with bilateral paramedian thalamic infarctions. These are vertical gaze palsy, impairment in memory, and coma. There can be akinetic mutism, drowsiness, and hypersomnolence. Patients presenting with these infarcts commonly have symptoms like unilateral weakness, cerebellar ataxia, movement debilitation, and oculomotor abnormalities. The timely detection and diagnosis are extremely important in cases of artery of Percheron infarction, as it remains often hidden for long, leading to irreversible consequences. The preferred imaging modalities for prompt diagnosis of artery of Percheron infarction are diffusion-weighted imaging (DWI) and fluid-attenuated inversion recovery (FLAIR) [3].

The main reasons responsible for delayed diagnosis are varied neurological symptoms at the time of presentation, difficulty in diagnosing artery of Percheron infarcts in acute computed tomography or MRI sometimes due to its small size, lack of awareness among physicians to suspect it prima facie. Various reports have been documented where initial imaging was not significant, suggesting that initial normal findings in MRI report cannot rule out the chances of this diagnosis. Although the condition is rare, each and every case can be used as the base for treating another case so that treatment methodologies can be rectified accordingly [3].

## Case presentation

A 57-year-old male presented to the casualty of this hospital with chief complaints of irrelevant talk associated with drowsiness for six hours. The patient was apparently alright six hours back. In the morning, he got up, did all his natural chores, and sat for breakfast at around 8.30 am; 15 min after completion of breakfast, he complained of dizziness and mild diplopia. Within the next hour, the relatives found that patient is not talking coherently and is getting drowsy. There was no history of fever, breathlessness, convulsions, or any associated complaints of headache and vomiting. Relatives then took the patient to a local practitioner and were referred to the institute by a private vehicle.

On examination in the emergency department, the patient was drowsy with a Glasgow coma scale of 9 (E2V3M4). He was afebrile, pulse was 65 beats per minute and regular, blood pressure was 120/70 mm of Hg, and respiratory rate was 18 breaths per minute. Jugular venous pressure was normal.

Central nervous system examination showed bilateral pupils of 3.5 mm sluggishly reacting to light. All other cranial nerves were intact. The patient was moving all four limbs with deep painful stimuli. Plantars were bilateral flexor. Cardiovascular system examination was normal, respiratory system and abdomen examination revealed no abnormality.

Laboratory investigations are mentioned in Table 1.

**Table 1:** Laboratory investigations of the case

Laboratory parameter	Measured value	Reference range
Hemoglobin	12 grams per deciliter	13.5 to 17.5 grams per deciliter
Total leukocyte count	4200/ mm^3^	4000 to 11000 cells/cubic millimeter of blood
Total platelet count	430,000 platelets per microliter of blood	150,000 to 450,000 platelets per microliter of blood
Serum urea	22 mg/dl	6 to 24 mg/dL
Serum creatinine	0.7 mg/dl	0.7 to 1.3 mg/dL
Serum sodium	138 meq/lit	135 and 145 milliequivalents per liter (mEq/L)
Serum potassium	4.1 millimoles per liter (mmol/L)	3.6 to 5.2 millimoles per liter (mmol/L)
Serum aspartate aminotransferase	40 units per liter	5 to 40 units per liter
Serum alanine transaminase	38 units per liter	7 to 55 units per liter (U/L)
Serum alkaline phosphatase	112 international units per liter	44 to 147 international units per liter (IU/L)
Serum albumin	4.1 g/dl	3.4 to 5.4 g/dL
Total bilirubin	1 mg/dl	< 1.2 milligrams per deciliter (mg/dL)
Total protein	6.4 g/dl	6.0 to 8.3 grams per deciliter (g/dL)

**Figure 1:**
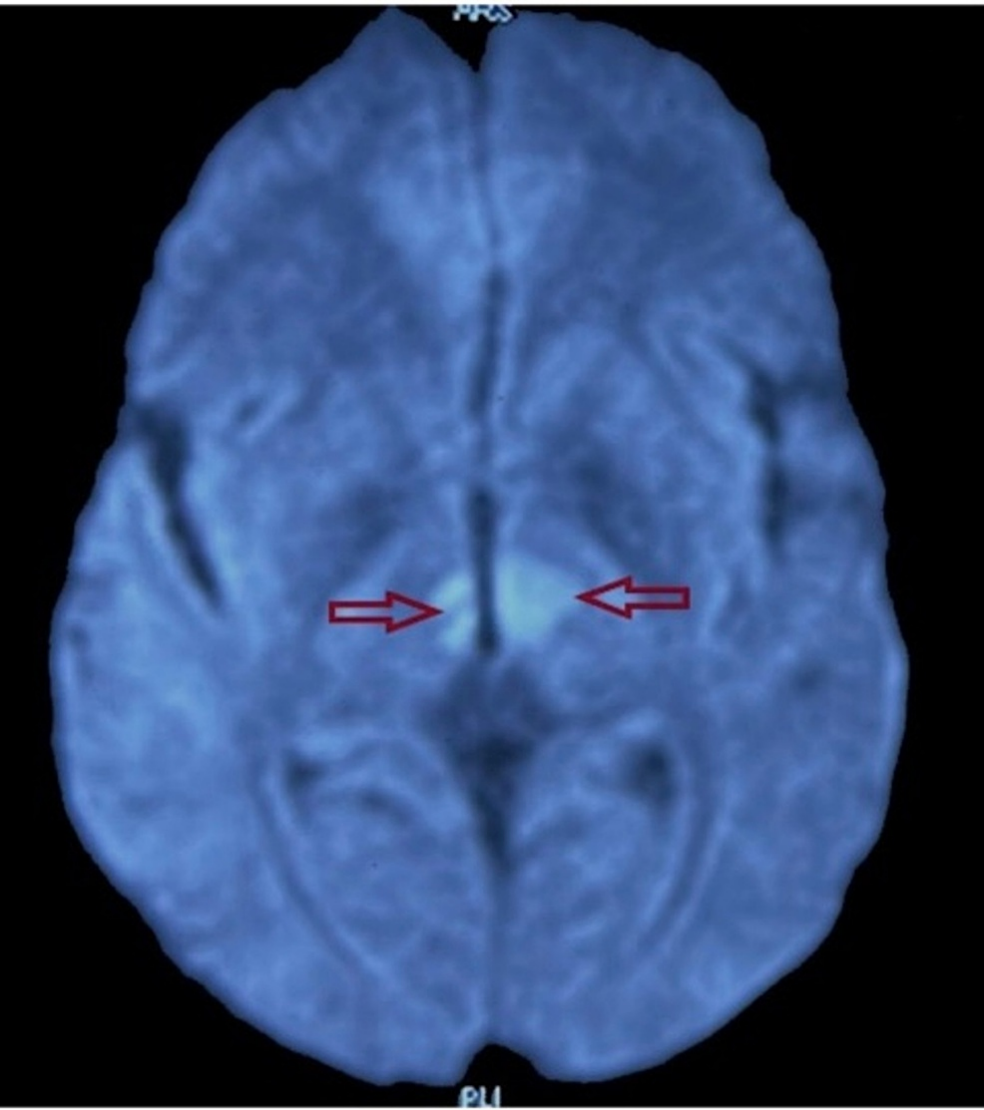
DWI axial section of the brain at the level of thalamus showing restricted diffusion in bilateral paramedian thalami consistent with the infarct of artery of Percheron (a variant of P1 segment of the posterior cerebral artery) DWI - diffusion-weighted imaging

**Figure 2:**
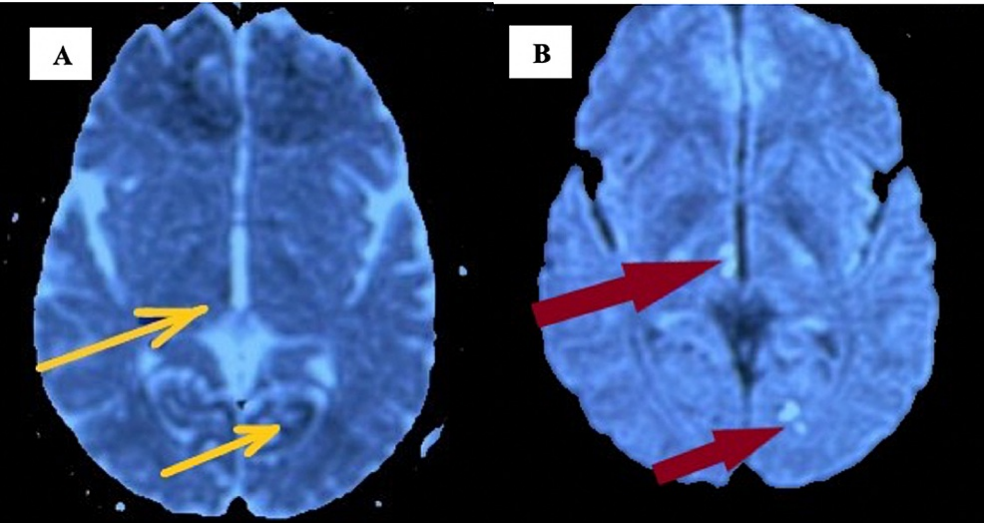
DWI axial section of the brain at the level of thalamus showing restricted diffusion in the right paramedian thalamus and left cerebellum (A), with corresponding dark signals on ADC (B) DWI - diffusion-weighted imaging; ADC - apparent diffusion coefficient

Electrocardiogram was within the normal limit. An emergency non-contrast computed tomography brain did not reveal any abnormality. MRI brain revealed bilateral thalamic, paramedian midbrain, subacute right side, acute left side artery of Percheron (variant of posterior cerebral artery P1 common trunk) territory non-hemorrhagic infarct (left >right; Figures 1-2).

Diagnosis of Percheron syndrome was made because the patient presented beyond the window of intravenous thrombolysis with tissue plasminogen activating factor (tPA). He was treated conservatively in the intensive care unit. A 2D echocardiogram was done, which was normal. Telemetry was negative for occult cardiac arrhythmias. 

The patient was treated with a dual antiplatelet regimen (tablet aspirin 150 mg plus tablet clopidogrel 75 mg per oral once a day). Two days after the hospital stay patient improved clinically and was fully awake and conscious. The patient was discharged on tablet dual antiplatelet therapy after five days. He was advised to come for follow-up after one month, after which the consideration of dual antiplatelet would be weighed versus a single tablet of aspirin.

## Discussion

**Figure 3:**
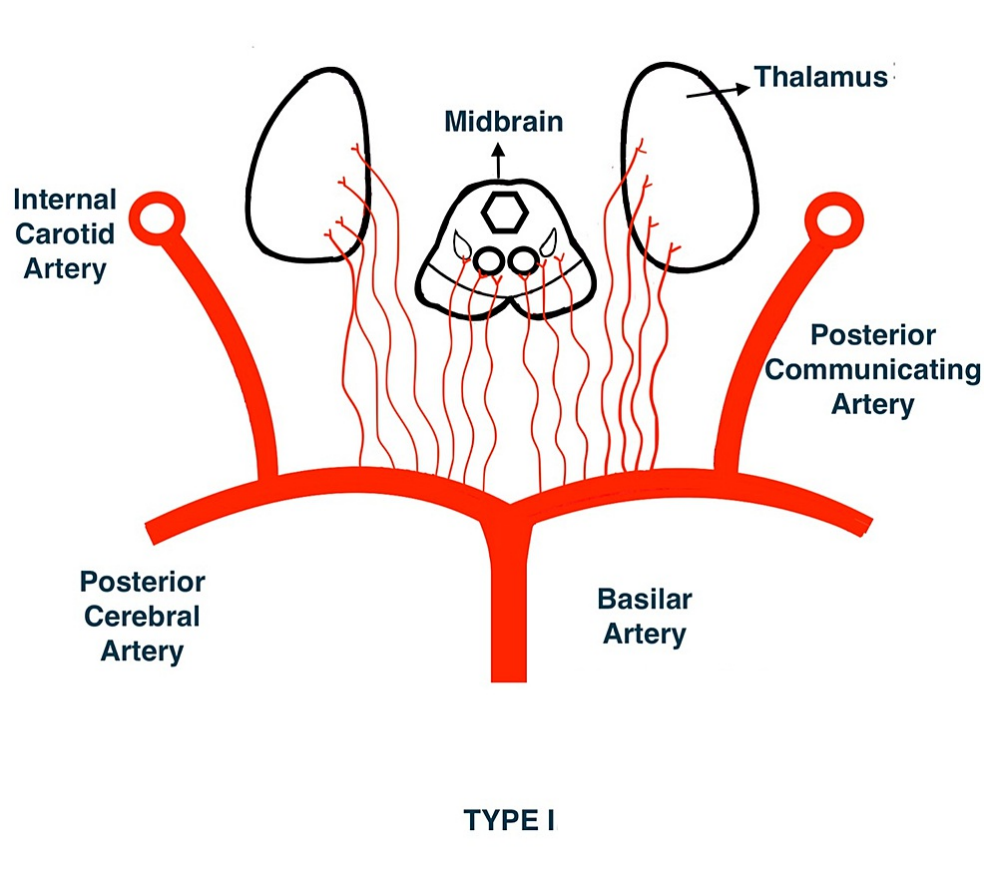
Type I variant of perforator supply of thalamus and the rostral midbrain

**Figure 4:**
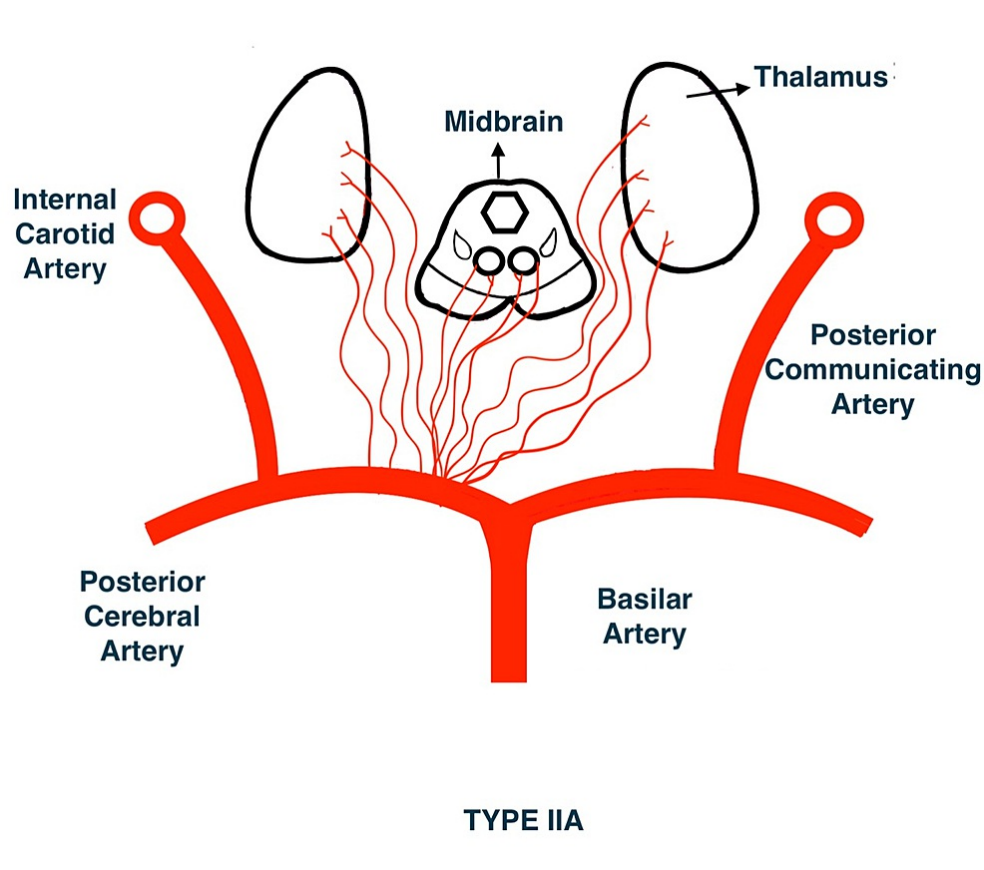
Type IIa variant of perforator supply of thalamus and the rostral midbrain

**Figure 5:**
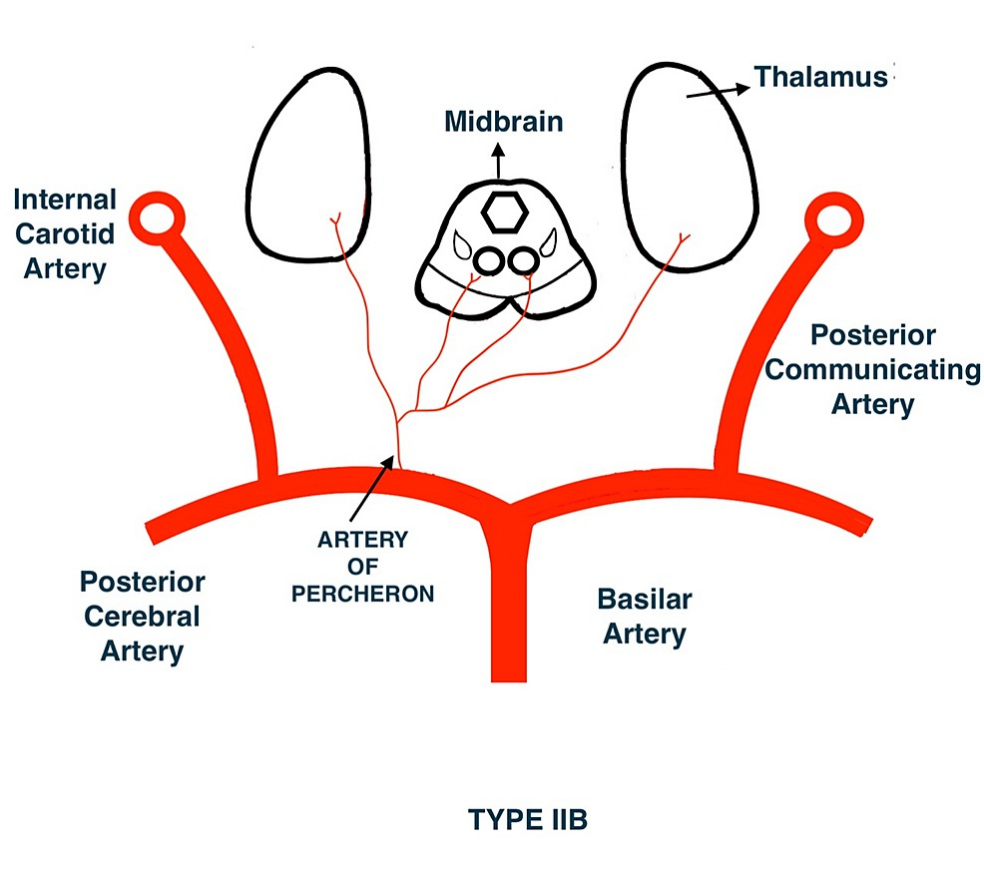
Type IIb variant of perforator supply of thalamus and the rostral midbrain

**Figure 6:**
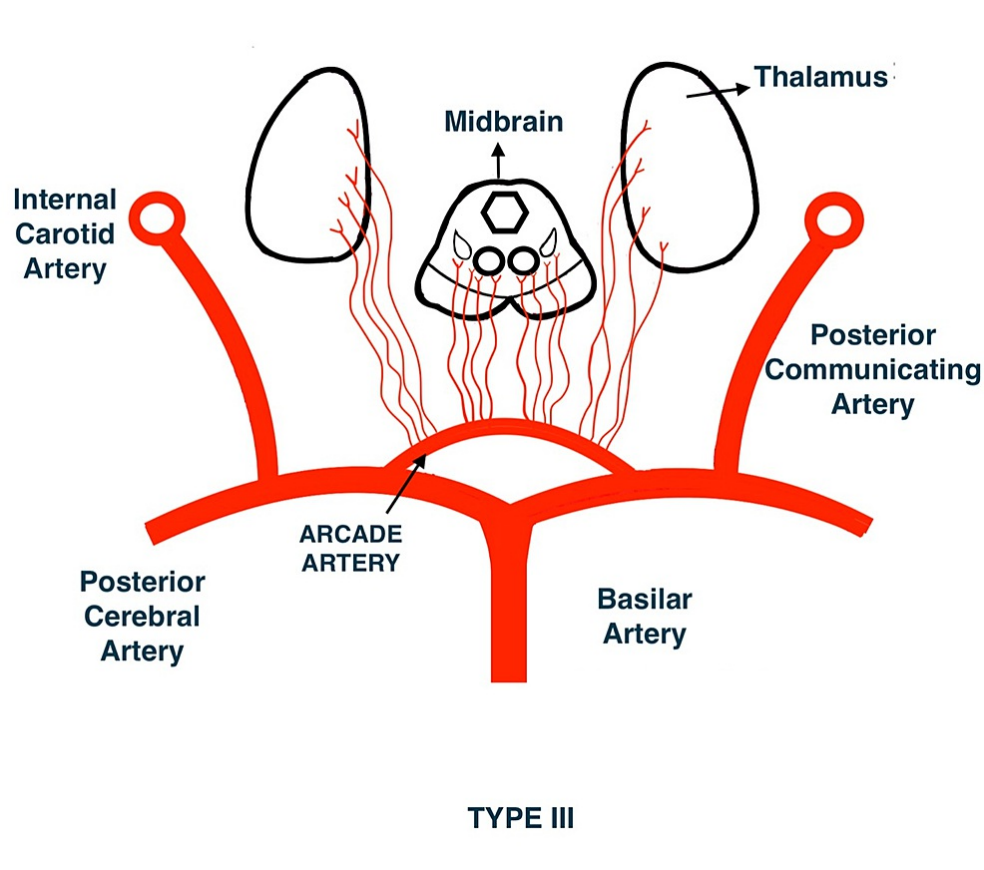
Type III variant of perforator supply of thalamus and the rostral midbrain

French neurologist Gerard Percheron described four variants of perforator supply of the thalamus and the rostral midbrain (Figures 3-6). The third variant (Type IIb) encompasses the artery of Percheron. This variant occurs in up to 25% of the population, accounting for a substantial portion of all thalamic infarcts [4].

Artery of Percheron arises from the P1 segment of a posterior communicating artery like a single trunk supplying both paramedian thalami. This trunk further branches to form bilateral perforating thalamic arteries as a rare variant. The precise prevalence of the artery of Percheron is unidentified, as its small size makes it tedious for recognition. It is the only artery to cause infarct at the paramedian thalamic region bilaterally and may involve the midbrain. Even though it is said to be rare to encounter, it has been visualized on MRI or MR angiography [5,6].

Thalamic infarcts are very scarce. It is accounted to be 11% of all infarcts involving the vertebrobasilar region. Different clinical presentations are mainly due to diverse and complicated anatomical structures and varying vascularity. Midbrain is sometimes involved due to overlapping vascularity. It becomes very critical to diagnose this infarction when the midbrain is involved along with paramedian thalami because of infrequent involvement and as the clinical presentations are not clear. Studies on the prevalence of artery of Percheroninfarction showed that it is 0.1-0.3%. [7,8].

Though the prevalent presentation of artery of Percheron infarct is drowsiness that may progress to coma, other associated features may be upward gaze palsy, abulia, memory loss when the hippocampus is involved. Cognitive abnormalities like emotional incontinence, hypersexuality, delusion, and delirium are also noted. When the midbrain is involved, then a distinct thalamopeduncular syndrome consisting of hemiplegia, cerebellar ataxia, and oculomotor palsy is seen [9,10].

Due to its varying anatomical presence and fluctuation in P1 segment size, the artery of Percheron infarction is a tough diagnosis to make. The investigations of choice for prompt diagnosis of artery of Percheron infarction are MRI diffusion-weighted imaging (DWI) and FLAIR MRI sequences. In an acute cerebral ischemic presentation, MRI permits visualization of the initial infarct and is commonly utilized as the primitive imaging modality in stroke centers [7].

Differential possibilities of bilateral thalamic lesions should be ruled out wherever necessary [11], as shown in Table 2.

**Table 2:** Differential diagnosis of the case

Conditions	Features	Radiological features
Creutzfeldt-Jakob disease	Infection with prion protein. Progressive dementia, myoclonus.	T2 is prolonged and diffusion is reduced in basal ganglia and thalami. Diffusion is also altered in the cortex known as cortical ribboning. A characteristic feature is the “pulvinar sign” a high-intensity signal in the pulvinar.
West Nile encephalitis and Japanese encephalitis	High fever, headache, neck stiffness, stupor, disorientation, convulsion, and coma.	Bilateral T2 hyperintensity in both thalami, basal ganglia, midbrain, and sulcus (leptomeningeal inflammation).
Wernicke's encephalopathy	Confusion, bilateral sixth nerve palsy, nystagmus, neuropathy, ataxia, anterograde amnesia.	MR signals are seen around the third ventricle, the periaqueductal region, mamillary bodies, and the tectal plate.
Cerebral venous sinus thrombosis	May involve bilateral thalami and basal ganglia. Usually seen in hypercoagulable states.	On MR images, abnormally hyperdense veins and clots in the sinuses can be observed as T1 hyperintensity on CT scans.
Posterior reversible encephalopathy syndrome (PRES)	Neurotoxic statepresenting secondary to the loss of autoregulation of the posterior circulation to acute hypertension leading to hyperperfusion injury and vasogenic cerebral edema in the posterior cerebral artery territory.	Characteristics of affected areas usually reflect vasogenic edema.
Leigh syndrome (mitochondriopathy)	Genetically heterogeneous mitochondrial disorder leading to respiratory failure and death in childhood.	Hyperintensity is seen in involved regions like basal ganglia, diencephalon thalami, and dentate nuclei on T2 weighted images.
Gangliosidoses (lysosomal disorders)	It has various lipid disorders caused by the accumulation of abnormal lipids also known as gangliosides.	The bilateral supratentorial white matter with hyperdense thalami seen on non-contrast CT.
Krabbe's disease	It is known as globoid cell leukodystrophy which is a lysosomal storage disorder leading to myelin turnover disorder.	Hyperdense areas that are symmetrically affecting the thalami, cerebellum, caudate nuclei, and the posterior limbs of the internal capsule along with the brainstem.
Wilson's disease	Copper metabolism disorder of autosomal recessive type.	There is no contrast enhancement. Early images show evidence of reduced diffusion, leading to restoration of normal diffusivity after necrosis and spongiform degeneration.
Non-Wilsonian hepatolenticular degeneration	Heretogeneousneurological disorder occurring secondary to acquired liver disease.	Intrinsic hyperintensity in globus pallidus, subthalamic region, and midbrain.
Thalamic glioma	It is diffuse low-grade astrocytoma occurring both in adults as well as children causing movement disorder.	Along with the expansion of both thalami, it is led by hyperintensity on T2 images along with hypointensity on T1.
Fahr disease	Neuropsychiatric abnormalities and parkinsonian or choreoathetotic movement disorder.	Calcification is seen in bilateral deep gray matter frequently involving globus pallidus other than putamen, caudate nuclei, thalami, and dentate nuclei.

AOP stroke remains a difficult scenario to diagnose due to variations in circulation. The prevalence of Type IIb AOP ranges from 4% to 18% of the population; however, this could be underestimated [12].

The conventional treatment for an acute ischemic stroke is dependent on various elements, consisting of time of start, the site of the lesion, and any thrombolytic contraindications. The optimum treatment for proximal cerebral artery occlusion is a recombinant tissue plasminogen activator given within 4.5 hours of its onset, followed by mechanical thrombectomy in less than six hours [13].

Long-term antiplatelet therapy of aspirin and clopidogrel is required when there is evidence of underlying cardioembolic events, or the etiology is cryptogenic.

## Conclusions

Ischemic stroke caused by the obstruction of the artery of Percheron is a rare type of ischemic stroke. Depending on the development of symptoms to the time of presentation, prompt diagnosis permits for better treatment. Early treatment with tPA and or mechanical thrombectomy positively affects patient outcomes. Otherwise, if a patient presents beyond the therapeutic window of thrombolysis, long-term antiplatelet therapy remains the cornerstone. AOP infarcts may present with a myriad of atypical symptoms that should be kept in mind during evaluation. Other differentials of bilateral thalamic lesions in neuroimaging should be entertained and ruled out wherever necessary.
